# Supplementary material for: Longitudinal assessment of changes in hair cortisol levels and associations with violence, poor mental health and harmful substance use among female sex workers in Nairobi, Kenya
Source: PLOS Glob Public Health. 2026 Jan 12;6(1):e0005013. doi: 10.1371/journal.pgph.0005013 (PMC12795377; doi:10.1371/journal.pgph.0005013)
Supplement: S1 Table — (DOCX) [file pgph.0005013.s001.docx]

**Longitudinal assessment of changes in hair cortisol levels and associations with violence, poor mental health and harmful substance use among female sex workers in Nairobi, Kenya.**

Mamtuti Panneh, MMSc^1^*, Tara Beattie, PhD^2^, Qingming Ding, PhD^3^, Rhoda Kabuti, MSc^4^, The Maisha Fiti study champions^4^, Polly Ngurukiri, BA^4^, Mary Kungu, BA^4^, Tanya Abramsky, MSc^2^, James Pollock, BScH^5^, Alicja Beksinska, BMBS^2^, Erastus Irungu, MSc^4^, Janet Seeley, PhD^2^, Helen A Weiss, DPhil^6^, Abdelbaset A. Elzagallaai, PhD^3^, Michael J Rieder, PhD^3^, Rupert Kaul, PhD^7^, Joshua Kimani, MBChB^4^, Mitzy Gafos, PhD^2**^, John Bradley, PhD^6**^

^1^Department of Infectious Disease Epidemiology and International Health, London School of Hygiene & Tropical Medicine, London, UK.

^2^Department of Global Health and Development, London School of Hygiene & Tropical Medicine, London, UK. Mamtuti.Panneh@lshtm.ac.uk.

^3^Robarts Research Institute, Schulich School of Medicine and Dentistry, Western University, London, ON, Canada.

^4^Partners for Health and Development in Africa, Nairobi, Kenya.

^5^Department of Immunology, University of Toronto, Toronto, Canada

^6^MRC International Statistics and Epidemiology Group, Department of Infectious Disease Epidemiology, LSHTM, London, UK

^7^Department of Medicine, University of Toronto, Toronto, Canada.

*Corresponding author: Email [Mamtuti.panneh@lshtm.ac.uk/ tutipanneh@gmail.com](mailto:Mamtuti.panneh@lshtm.ac.uk/%20tutipanneh@gmail.com)

** Authors contributed equally

**Table S1.** Comparison of the characteristics of study participants in this follow-up study (N=285) with the study participants at baseline (425) and the HIV-negative participants in The Maisha Fiti study who were excluded at baseline (N=321)

| Characteristic | Total 285  N(%) | Total 425  N (%) | Total 321  N (%) |
| --- | --- | --- | --- |
| Age |  |  |  |
| <25 | 90 (18.6) | 119 (16.1) | 81 (14.3) |
| 25-34 | 101 (42.2) | 155 (42.5) | 131 (46.8) |
| 35+ | 94 (39.3) | 151 (41.4) | 109 (38.9) |
| Age at first sex |  |  |  |
| </=15 | 88 (30.5) | 130 (30.4) | 122 (39.0) |
| 16-17 | 97 (33) | 148 (33.8) | 88 (25.5) |
| 18+ | 97 (36.5) | 143 (35.80) | 108 (35.5) |
| Literacy |  |  |  |
| illiterate | 35 (12.9) | 61 (15.3) | 49 (16.59) |
| literate | 250 (87.1) | 364 (84.7) | 272 (83.41) |
| Religion |  |  |  |
| Catholic | 118 (40.8) | 172 (39.7) | 114 (35.2) |
| Protestant | 144 (51.3) | 220 (52.8) | 165 (53.2) |
| Muslim/others/none | 23 (7.9) | 33 (7.5) | 40 (11.6) |
| Socio-economic status |  |  |  |
| Lower/lower middle | 105 (35.8) | 161 (37.2) | 138 (41.9) |
| middle | 53 (17.9) | 76 (17.4) | 61 (18.9) |
| upper middle/upper | 127 (46.3) | 188 (45.4) | 122 (39.2) |
| Total number of ACEs reported |  |  |  |
| 0 to 4 | 79 (27.3) | 110 (25.4) | 86 (27.6) |
| 5 to 8 | 161 (57.1) | 246 (58.6) | 182 (56.0) |
| 9 to 12 | 45 (15.6) | 69 (16.0) | 53 (16.4) |
| Marital Status |  |  |  |
| Single | 91 (27.7) | 125 (26.5) | 94 (26.2) |
| Married or cohabiting | 16 (6.1) | 28 (7.0) | 29 (9.1) |
| Separated/divorced /widowed | 178 (66.3) | 272 (66.5) | 198 (64.8) |
| Number of Children* |  |  |  |
| None | 17 (4.8) | 24 (4.8) | 22 (2.8) |
| one to two | 188 (69.3) | 274 (66.5) | 216 (69.8) |
| 3+ | 60 (25.9) | 101 (28.6) | 74 (27.5) |
| Number of household dependents |  |  |  |
| 0 | 62 (19.3) | 82 (17.8) | 58 (16.7) |
| 1 | 78 (25.8) | 117 (25.4) | 72 (21.2) |
| 2+ | 145 (54.8) | 226 (56.8) | 191 (62.1) |
| Social support |  |  |  |
| No | 89 (30.6) | 119 (27.5) | 88 (28.0) |
| Yes | 196 (69.4) | 306 (72.5) | 233 (72.0) |
| Place of selling sex |  |  |  |
| Lodge/hotel/rented room/home | 272 (96.8) | 406 (97.0) | 312 (97.2) |
| Public places | 9 (3.2) | 13 (3.1) | 9 (2.9) |
