# Supplementary material for: Longitudinal assessment of changes in hair cortisol levels and associations with violence, poor mental health and harmful substance use among female sex workers in Nairobi, Kenya
Source: PLOS Glob Public Health. 2026 Jan 12;6(1):e0005013. doi: 10.1371/journal.pgph.0005013 (PMC12795377; doi:10.1371/journal.pgph.0005013)
Supplement: Attachment — Submitted filename: Rebuttal letter longitudinal cortisol_Mamtuti_30 May 2025.docx [file pgph.0005013.s004.docx]

Dear PLOS Global Public Health

**Ref**: PGPH-D-24-03042

Longitudinal assessment of changes in hair cortisol levels and associations with violence, poor mental health and harmful substance use among female sex workers in Nairobi, Kenya.

We thank the reviewers for their thorough and constructive comments on our manuscript. We have revised the manuscript according to their suggestions and have provided the details in the table below.

We look forward to hearing from you.

Kind regards,

Mamtuti Panneh.

| **Reviewer’s comment** | **Author’s comments** | **Relevant Text** |
| --- | --- | --- |
| **Reviewer 1** |  |  |
| This is a very nice manuscript exploring changes in hair cortisol concentrations in FSWs who are HIV negative and participating in a study in Kenya. The manuscript is interesting and well written, though I have some suggestions to improve it. | Many thanks for your comments. |  |
| Abstract | | |
| 1. Line 42 – how long after the baseline visit was the endline visit? | Thank you for this useful comment.  Endline data collection started 12 months after the baseline. I have now revised the sentence to include this information as follows:  ‘We used baseline and endline data from the Maisha Fiti study of FSWs in Nairobi, collected 12 months apart’. | Lines 41-42 |
| 2. Line 51 – the term “trajectories” is vague – please describe more fully | Thank you for the comment. We have now added further description as follows:  ‘Multivariable linear regression models were used to assess the associations between the trajectory of exposure to violence, mental health problems and harmful substance use from baseline to endline and the change in HCC levels at endline. Each exposure trajectory was categorised into three levels: present at neither time point, present at baseline only, and present at endline (with or without baseline). | Lines 46-50 |
| 3. There is no indication of the hypothesised direction of an effect nor of the results – the term “change” is used | Thank you for pointing out this important oversight. We have now revised the sentence to include the direction of change in the results, as follows:  There was evidence of associations between the trajectories of physical violence (p-value=0.007) and physical and/or sexual violence (p-value = 0.048) with decreased HCC at endline | Lines 53-55 |
| Introduction | | |
| 1. Nicely written overall. | Many thanks for the comment |  |
| 2. Lines 133-146 do not hypothesize a main exposure variable, nor hypothesized directionality of changes. Did the authors expect increases or decreases? Or given the prior discussion about hyper- and hypo-cortisolism, maybe it is exploratory? This should be stated up front. | Thank you for this valuable feedback. As the first longitudinal study conducted among FSWs and given the inconsistencies in the literature regarding associations between violence, mental health and harmful substance use with HCC, this study aimed to explore how cortisol levels change over time in relation to these exposures. We have revised the text to better reflect this, as follows:  ‘In this paper, we used the baseline and endline data to examine whether hair cortisol levels change over time from baseline to endline and explore if hair cortisol levels changed based on the trajectory of exposure to different types of violence, mental health problems and harmful alcohol and other substance use among HIV-negative FSWs in Nairobi from baseline to endline’. | Line 142 |
| **Methods** | | |
| 1. Please note the standardized scales within the text. I had to go to Table 1 to find it all. | Many thanks for this useful feedback. We have now added standardised scales within the text. | Lines 276-296 |
| 2. Lines 256-260 – define alcohol and substance use “problems” | We apologise for this oversight. We have now defined harmful alcohol and other substance use as follows:  ‘The WHO ASSIST (Alcohol, Smoking and Substance Involvement Screening Test) tool was used to assess alcohol risk (cut-off scores: low risk 0–10; moderate risk 11–26; high risk 27 +) and other substance use risk (cannabis, cocaine, amphetamines, hallucinogens, sedatives and inhalant) in the last three months (cut-off scores: low risk 0–3; moderate risk 4–26; high risk 27 +). Harmful use of alcohol and other substances was defined as scoring ≥ 11 and   ≥ 4, respectively, on the WHO ASSIST tool’ | Lines 289-294 |
| 3. Give dates of the study, and time elapsed between the 2 visits | Thank you so much for the feedback. The time between the 2 visits was 12 months, and we did indicate the dates of the study in the first paragraph in the methods section as follows:  ‘*Baseline data were collected from 14 June to 13 December 2019 before the onset of the COVID-19 pandemic, while endline data were collected between 11 June 2020 and 29 January 2021 after the COVID-19 lockdown in Kenya’* | Lines 157-159 |
| 4. Given the large number of predictor variables of interest, would it be appropriate to consider using multiple comparisons for the p-values? | Thank you for this thoughtful comment. We are aware that multiple comparisons can be an issue, but to our knowledge, there is no universal agreement on how to handle it. We believe that in an observational study like this, it is reasonable not to adjust p-values for multiplicity for the following reasons: 1) This type of study, in which many associations are assessed, is naturally of an exploratory nature. This is distinct from a clinical trial in which it is vital to take multiplicity of comparisons into account. 2) Although there is no universal standard for this type of study, we believe the vast majority do not adjust p-values. And 3) The Bonferroni correction (and other similar methods) are highly conservative and are far too stringent for a study like this that examines multiple exposures, making it almost impossible to achieve significance. There is no reasonable method available for taking multiplicity into account for p-values. |  |
| Results | | |
| 1. Line 316 – Give breakdown of who did not have complete data from the parent study, and reasons why excluded, and examine if group differed. | Thank you for the comment. The main reason for the exclusion of participants in this study is the unavailability of cortisol data due to poor sample quality, which we explained in the Methods section Lines 249-255.  We have now added a supplementary document comparing the characteristics of study participants with those of the parent study. We have also indicated in the results section the text below:  ‘The characteristics of these study participants are similar to the 425 HIV-negative participants with cortisol results at baseline, the 321 HIV-negative participants excluded at baseline from the Maisha Fiti study, and the overall baseline sample of the Maisha Fiti study published elsewhere (4). **Supplementary Table 1** presents a comparison of the characteristics of participants included in this study (N=285), the full baseline sample (N=435), and the HIV-negative participants excluded at baseline.’ |  |
| 2. Line 347 – Is it possible to present mean paired differences | We thank the reviewer for this suggestion. There is a balance between including information that could be useful and brevity. We have included an extensive set of results, and here we do not believe the addition of this extra information would justify the space taken up in what is already quite a long paper. |  |
| 3. Throughout – the term “never” threw me off. It is only for those 2 data points, so maybe make the name more informative, like “Neither timepoint”. | Thank you for picking up this important oversight. We agreed that the term ‘never’ can be misleading. We have now changed it to ‘neither timepoints' across the document as suggested.  We have now made the categorisation clearer in the methods section as follows:  ‘The trajectories of all time-variant exposures were categorised into three levels: present at neither timepoint, present at baseline only (baseline: yes; endline: no), and present at endline (with or without baseline). |  |
| 4. Similarly, the term “baseline only” in essence means a decrease in the variable (or absence of) from baseline to follow-up, so I’d call that group “Decreased” or “Baseline: yes; Endline: no”. | Thank you for the comment. As stated above, we have now made this clear in the methods section by showing the meaning of baseline only (Baseline: yes; Endline: no). |  |
| 5. “Endline (with/without baseline)” was more clear, but it would be clear to call them “Increased or sustained”. | Many thanks for this comment. While we agree that the term "increased" or "sustained" could be a reasonable way to label the category, we chose to retain the original name. This is because some participants who were exposed at endline had no exposure at baseline, and the suggested terms may not fully capture that pattern. |  |
| 6. I have a hard time interpreting the comparisons to the reference category, i.e. low risk at both timepoints. I would think the important comparison would be between sustained high risk and decreased risk. This is related to my prior comment about the lack of hypothesis, and the vague term trajectories. If these comparisons were made, the significant findings (which were all in comparison to the no risk at either time point), which is hard to interpret. | Thank you for this thoughtful comment. We apologise for any confusion in the interpretation of our findings.  This was an exploratory longitudinal study, aiming to examine how changes in exposure to stress-related factors such as violence, poor mental health, and substance use over time were associated with changes HCC. The relationship between stress and HPA axis activity is complex and not yet fully understood, particularly in vulnerable populations such as FSWs. Both elevated and suppressed HCC levels have been observed in response to stress, depending on several factors, including the timing, severity and duration of exposure. Also, there is no universally accepted clinical cutoff for defining abnormal HCC levels.  Given this, our analysis was intentionally exploratory. We emphasised that lower HCC does not necessarily indicate lower stress or better HPA-axis function. In some cases, low cortisol may reflect more severe or prolonged dysregulation, known as the attenuation effect, where chronic exposure leads to a blunted cortisol response.  To explore these patterns, we classified participants based on their exposure to violence over time (e.g., baseline only, endline, or neither) and used the group with no exposure at either time point as the reference. We found that women who experienced violence either at baseline or endline had lower HCC at endline compared to the reference group. These findings are consistent with some prior studies showing reduced cortisol among individuals recently or chronically exposed to violence as emphasised in the discussion section.  We have revised the Results and Discussion sections to improve the clarity and interpretation of the findings. | Lines 395-409 (results section) Lines 464-489 (Discussion section) |
| 7. Line 385 – add the word “receiving” before counselling to remind readers that this was not a randomized state | Many thanks for picking up this important oversight on our part. We have now added the word ‘receiving’ before counselling throughout the manuscript. |  |
| 8. Line 389 – in talking about the interaction, give the p-value. The stratified GMRs do not show the test for interaction. | Many thanks for this useful comment. We have now presented the P-values of the interaction test in text as follows:  ‘When we examined the interaction of counselling, there was evidence that the association between the trajectories of exposure to physical violence (p-value=0.002) and physical and/or sexual violence (p-value =0.009), with decreased HCC at endline, were modified due to receiving counselling (**Table 4**)’ | Lines 407-409 |
| **Discussion** | | |
| 1. Lines 509-510. Move this to the results. | Thank you for the comment. Since this information is already included in the Results section (**Lines 343-348**), we have now deleted it from the Limitations section. |  |
| 2. Lines 467-473 This sounds like a mediation claim, yet mediation was not analyzed, so the claim is unfounded. | We do not understand this point from the reviewer. We have looked at the association between exposures (mental health problems or harmful substance) with an outcome (HCC levels). No mediating variables were considered and we have made no claim about mediation. |  |
| 3. Given the lack of a hypothesized direction of the HCC results, the discussion is somewhat muddled. Framing it either as exploratory, or giving a clear hypothesis would help the discussion flow. | As mentioned earlier, this study was exploratory, and we have now indicated that in the text to guide the reader. |  |
| 4. I have a hard time interpreting the results given my comment #6 above. | We apologise for any confusion caused in interpreting the findings and have revised the manuscript to improve clarity. |  |
| **Reviewer 2** | | |
| A) OVERALL COMMENTS: This paper addresses a highly relevant and timely topic with clear public health significance, particularly in the context of HIV epidemic control in sub-Saharan Africa, where this key population plays a central role. Indeed, from the body of literature available, it bridges the gap related to unavailability of longitudinal understanding of HCC levels in relation to the specified exposures of interest.  However, as a longitudinal or follow-up study published in Discover Mental Health (August 2024), it is standard practice in the research publication landscape to build on previously published work without repeating large sections from the original paper.  As noted under specific sections — including study design and sampling (Lines: 161–177), study variables (Lines: 242–253), and statistical analysis (from Line: 286 onward) — several sentences appear nearly identical to those in the original publication. While it is commendable that the original study has been cited, I recommend a review of these sections to minimize repetition and improve conciseness. Streamlining this content will not only maintain academic integrity but also enhance the readability of the manuscript.  Otherwise, the manuscript is technically sound with the inferences being supported by data. The paper represents rigorous research both methodologically and from an ethical perspective. Also, the authors have made all data underlying the findings fully in line with PLOS GPH’s requirements. | Thank you for your valuable feedback. We have revised the manuscript accordingly, including editing the methods section to eliminate any repetition of exact wording from our original manuscript. | Lines 166-178  Lines 246-248 |
| In accordance with my review, the manuscript has been adequately presented in an intelligible fashion and written in standard and understandable English. | Many thanks! |  |
| **Introduction** | | |
| Given the established biological and epidemiological links suggesting that both COVID-19 infection and the broader psychosocial disruptions associated with the pandemic may contribute to chronic stress and dysregulation of the hypothalamic-pituitary-adrenal (HPA) axis, the author should consider enhancing the Introduction by including a brief discussion on the potential role of acute and long COVID-19 in elevating hair cortisol concentration (HCC). This addition is particularly important, as COVID-19 is later integrated into the conceptual framework. Although the study did not directly measure COVID-19 exposure or infection status, articulating this theoretical connection early in the manuscript would strengthen the rationale for its inclusion and provide a clearer conceptual foundation for the subsequent analysis (considering the fact that the original paper did not have COVID-19 included in the conceptual framework). | Thank you for this valuable feedback. We agree that including a brief discussion of the potential impact of the COVID-19 pandemic on cortisol levels in the Introduction would help clarify why the pandemic was considered an important variable in this study. However, since we were unable to directly measure the pandemic’s direct and indirect effects, we have instead included a brief explanation in the Methods section to justify the inclusion of COVID-19 as a possible factor influencing cortisol levels, as outlined below:  ‘The effect of the COVID-19 pandemic on cortisol levels was included in the framework due to its widespread stress-related impact. For example, in response to the pandemic, a prospective study in the UK reported a substantial increase in cortisol levels among adults (39). Evidence of higher cortisol levels among patients with severe COVID-19 infections and lower levels among those with long-term COVID-19 infections has been reported (40, 41).’ | Lines 215-220 |
| 2) METHODS: | | |
| a) Study Design and Sampling Line 176: Since both in the original paper (Reference No. 37) and this current one the analysis focused solely on the HIV-uninfected participants, this statement might make the reader think there were differences between the two. As the authors address the earlier highlighted recommendation, this specific statement should be looked at as well. | Thank you for pointing this out — we agree with your observation. We have now deleted line 176, as the focus on HIV-negative women was already clearly stated in the Introduction. |  |
| b) Data Collection: Behavioural-biological survey • Line 188: The author refers includes in the list of time-invariant sociodemographic factors, age, marital status, and number of children. While in an infinitesimal period of time this might be true, in the actual sense they are liable to change. • Line 192: I would like to point out an editorial comment that requires correction. The author mentions that "blood samples were used to test for syphilis using rapid plasma ‘regain’ assay". I believe this is a typo; it should read plasma ‘reagin’ assay. Please revisit and rectify accordingly. | Thank you for your valuable comment. We agree that sociodemographic variables such as age, marital status, and number of children can change over time. However, given the relatively short time frame between baseline and endline in our study, we considered these changes to be minimal or unlikely to significantly affect the analysis. Therefore, we treated these variables as time-invariant for the purposes of this study.  About the typo, thank you so much for picking it up. We have now corrected it in Line 193 |  |
| 3) DISCUSSION |  |  |
| a) Strengths and Limitations Line 493: While the author notes the use of validated tools to assess the main exposures, it is important to acknowledge a specific limitation of the WHO ASSIST tool - namely, its inability to accurately capture the quantity or dose of alcohol and substance use.  A related manuscript by the MAISHA team, published in Global Public Health (January 2024), reported that, in response to the COVID-19 pandemic, “To cope they [i.e., FSW] skipped meals, reduced alcohol use and smoking, started small businesses to supplement sex work or relocated to their rural home”.  In such contexts, the WHO ASSIST may detect a reduction in reduced frequency of alcohol and other substance use but not necessarily a reduction in quantity consumed per occasion. This distinction is critical, as reductions in frequency accompanied by continued high-dose consumption may still pose significant physiological risks, including elevated hair cortisol concentration (HCC). Therefore, alternative tools such as the WHO AUDIT (Alcohol Use Disorders Identification Test), which includes questions on both frequency and quantity of alcohol use, may have been more appropriate at least for measuring alcohol consumption.  Therefore, it is recommended that the author explicitly acknowledge this limitation in the manuscript to provide a more nuanced interpretation of the findings. | We sincerely appreciate this insightful and constructive feedback. We agree that the WHO ASSIST tool is limited in capturing the quantity of alcohol and other substances use and have now added it in the limitation section as shown below:  ‘However, the WHO ASSIST tool used to evaluate alcohol and other substance use risk has limitations, particularly in capturing the quantity consumed. For instance, a reduction in harmful use accompanied by continued high-dose consumption on individual occasions could still influence cortisol levels. Alternative tools, such as the WHO AUDIT, which assess both the frequency and quantity of alcohol consumption, may have been more suitable for capturing patterns relevant to cortisol levels’. | Lines 516-522 |
